# Supplementary material for: Correction: Towards a deeper understanding of parenting on farms: A qualitative study
Source: PLoS One. 2018 Sep 6;13(9):e0203842. doi: 10.1371/journal.pone.0203842 (PMC6126865; doi:10.1371/journal.pone.0203842)
Supplement: S2 File — (PDF) [file pone.0203842.s002.pdf]

PLOS ONE

RESEARCH ARTICLE

Towards a deeper understanding of parenting on farms: A qualitative study

Valerie Elliot[1], Allison Cammer[1,2], William Pickett[3], Barbara Marlenga[4], Joshua Lawson[1,5], James Dosman[1,5], Louise Hagel[1], Niels Koehncke[1,5], Catherine Trask[1,5]*, for the Saskatchewan Farm Injury Cohort Team¶

1 Canadian Centre for Health and Safety in Agriculture, College of Medicine, University of Saskatchewan, Saskatoon, Saskatchewan, Canada, **2** College of Pharmacy and Nutrition, University of Saskatchewan, Saskatoon, Saskatchewan, Canada, **3** Department of Public Health Sciences, Queen's University, Kingston, Ontario, Canada, **4** National Children's Center for Rural and Agricultural Health and Safety, Marshfield Clinic Research Foundation, Marshfield, Wisconsin, United States of America, **5** Department of Medicine, College of Medicine, University of Saskatchewan, Saskatoon, Saskatchewan, Canada

¶ Membership of The Saskatchewan Farm Injury Cohort Team is provided in the Acknowledgments.
* cmtrask@gmail.com

Check for updates

OPEN ACCESS

Citation: Elliot V, Cammer A, Pickett W, Marlenga B, Lawson J, Dosman J, et al. (2018) Towards a deeper understanding of parenting on farms: A qualitative study. PLoS ONE 13(6): e0198796. https://doi.org/10.1371/journal.pone.0198796

Editor: Lise Olsen, University of British Columbia Okanagan, CANADA

Received: April 26, 2017

Accepted: May 27, 2018

Published: June 13, 2018

Copyright: © 2018 Elliot et al. This is an open access article distributed under the terms of the Creative Commons Attribution License, which permits unrestricted use, distribution, and reproduction in any medium, provided the original author and source are credited.

Data Availability Statement: Data cannot be shared publicly as the University of Saskatchewan Research Ethics Board has forbidden the public release of information that could personally identify study participants. For data requests and to obtain more information regarding ethics certificate number Beh 12-265, contact the University of Saskatchewan Research Ethics Office (ethics.office@usask.ca, +1 (306) 966-2975, https://vpresearch.usask.ca/researchers/ethics1.php).

Funding: This research was funded by the Canadian Institutes of Health Research Operating

Abstract

Background

Children living on farms experience exceptionally high risks for traumatic injury. There is a large body of epidemiological research documenting this phenomenon, yet few complementary studies that have explored the deep underlying reasons for such trends. Fundamental to this is understanding the decision-making processes of parents surrounding their choice to bring children, or not, into the farm worksite.

Objectives

To (1) document farm parent views of the risks and benefits of raising children on a family farm, and, (2) understand more deeply why children are brought into the farm worksite.

Methods

Interviews were conducted as part of a larger cohort study, The Saskatchewan Farm Injury Cohort. Subsequent to an initial mail-out question focused on parental decision-making, 11 semi-structured telephone interviews were conducted with rural Saskatchewan farm parents. Interviews were digitally recorded and transcribed verbatim, then thematically analyzed using interpretive description methodology.

Findings

This parental decision-making process on farms fundamentally involves weighing the risks vs. benefits of bringing children into the worksite, as if on a balance scale. One side of this scale holds potential risks such as exposure to physical and chemical farm hazards, in the absence of full supervision. The other side holds potential benefits such as meeting family needs for childcare, labour, and family time; building work ethic and pride; and the positive impacts of involvement and responsibility. Decision-making 'tips the scales', in part

Grant 200109MOP-230156 – PH1-CEDA-56847 "Saskatchewan Farm Injury Cohort – Phase 2". CT was funded by a Canada Research Chair in Ergonomics and Musculoskeletal Health. JL was funded by a Canadian Institutes of Health Research and Lung Association of Saskatchewan New Investigator Award. The authors declare that they have no competing interests.

Competing interests: The authors have declared that no competing interests exist.

dependent upon parental perceptions of the risk-benefit trade-off. This 'perceptual lens' is influenced by factors such as: the agricultural way of life, parents' prior knowledge and past experience, characteristics of children, and safety norms.

Conclusions

This novel qualitative study provides deep insight into how Saskatchewan farm parents approach a fundamental decision-making process associated with their parenting. The proposed model provides insight into the etiology of pediatric farm injuries as well as their prevention.

Introduction

Children are vulnerable to traumatic injury on farms [1,2], yet the prevention of pediatric farm injuries has proven and encounter these hazards be a highly complex issue for both parents and safety professionals. Farms are unique settings that typically integrate both a hazardous worksite and a family home. Parental decisions made about children on farms must therefore involve considerations of occupational safety and health, as well as more general aspects of health in the developing child.

A pre-requisite for child injury on a farm is exposure to hazards: machinery-related injury is subsequent to exposure to machinery, livestock injuries are subsequent to exposure to livestock, and grain engulfment occurs after exposure to grain. This is intuitive and likely readily understood by farm parents and guardians. However, the persistence of child injury indicates that children continue to be in the farm work environment and encounter these hazards, even with safety campaigns promoting knowledge and mitigation of these hazards, such as the North American Guidelines for Children in Agricultural Tasks (NAGCAT) [3]. It would appear that current prevention efforts are not resonating with this population. There are clearly other motivating factors involved in parents' decision-making around bringing children into the farm worksite that are not well understood. Although there is a large body of research spanning decades that documents the epidemiology of child injuries on farms [1,2,4], fewer studies have deeply explored the perspectives and decision-making processes of farm parents regarding bringing children into farm worksites. For example, Green interviewed (man-woman) farm couples to investigate gendered perceptions of farm safety risks, safety strategies, and influencing factors [5]. In terms of parents' perceptions, Ashida et al. [6] have investigated parents' motivational barriers to using NAGCAT guidelines using protection motivation theory. Zepeda and Kim [7] conducted focus groups of parents on dairy farms to identify perceived benefits to children working on farms. Lee et al. [8] have taken a theoretical approach to the issue of child farm safety by applying the socio-ecological model's 'spheres of influence'. Nilsson used interviews with farm parents to identify themes of risks to children on farms, children's farm tasks, and farm risk education for children [9]. Despite this preliminary work in the area, there remains no consensus model for how parents decide to bring children into a farm work environment.

Objectives

As part of a longstanding effort to research the causes and consequences of farm injuries to children, there was a unique opportunity to conduct an in-depth exploration of parental

decision-making surrounding children's exposure to the farm worksite. A primary motivation for this work is long-term efforts to effectively translate best practices for childhood farm safety and develop intervention strategies that consider the "voices" and motivations of farm families in making safety decisions and are therefore more likely to gain traction within the farm community. The hope is that a better understanding of parents' decision-making process will facilitate the participation of farm communities in population-based strategies to minimize injury and optimize health in their children.

To serve this long-term aim, the objectives of the present study were to identify and explore farm parent views of the risks and benefits of raising children on a family farm and to understand why children are brought into the farm worksite. The ultimate application of this work will be to determine appropriate prevention strategies that would be accepted by farm parents.

Methods

Study location and history

This study was conducted in rural Saskatchewan, Canada as part of a larger study, the Saskatchewan Farm Injury Farm Cohort Study (SFIC) [10]. This study involved a large cohort of rural families and was designed with the purpose of studying farm injuries and their determinants. SFIC Phase 2 included the initiation of a specific cohort focused on children living in rural areas [11], not present in SFIC Phase 1. All work completed as part of this project was approved by the Behavioral Research Ethics Board at the University of Saskatchewan.

Study population and recruitment

The baseline for the Phase 2 child cohort was conducted in January to April 2014. We received permission from 46 schools (participation rate: 71.8%) representing 9,300 students to distribute study packets through the schools to parents for self-completion. A total of 1129 families, representing useable information on 2328 children took part. Study packets included a questionnaire asking about children's engagement in farm activities, injury occurrence in the past calendar year, general health status, health behaviours, and socio-demographic characteristics. In addition to this, questionnaires included an item asking permission to send follow-up surveys. Over the next two years, we conducted four follow-up surveys with those families who expressed interest.

To better understand determinants of children's farm injury and reasons for decisions to include children in farm work areas, we asked parents to describe their reasoning and thought process when bringing a child into the farm work environment. To accomplish this, we included an open-ended question on one of the postal follow-up surveys (January 2016) to explore parental reasoning and perspective on bringing children into the farm worksite. This written question included an opportunity to provide contact information if they were interested in participating in a telephone interview. Both these requests for information were embedded in the follow-up survey of the larger postal study of child farm injuries. The current manuscript focuses on a small sub-sample of the cohort study participants who completed the telephone interviews conducted after the open-ended written responses.

Data collection

In January 2016, our open-ended question asked parents to recall a situation where an adult, responsible for the care of a child, brought a child into the worksite while performing farm work. This question was embedded in a postal survey on farm injury among children and sent

to 845 households. Parents were asked to describe reasons that contributed this decision, as follows:

> "We ask you to recall a situation where an adult responsible for the care of a child (you or someone else) brought a child along while doing farm work. This could be, for example, during work involving farm machinery, work with farm animals, routine chores, etc. What do you think were some of the reasons that contributed to the decision to bring the child into the farm work area? Please write your responses in the box below."

Information collected from the 94 open-ended question survey responses were analyzed thematically by members of the research team (VE, AC, CE, WP, LH, CT), resulting in four main categories and subcategories as follows: *teaching* farming skills, safe practices, and work ethic; *needs* for labour, child care, and family time; perpetuating *farm culture* including traditions, culture, and values; and *health benefits* of being outdoors. These findings were used to develop a semi-structured interview guide for the telephone interviews. The guide included seven questions on demographics (sex/age of parent; sex/age of children; type of farm operation; years farming; how long lived on farm) and seventeen semi-structured questions/prompts designed to facilitate an in-depth discussion rather than prescriptive questioning on decision-making and risks/benefits of farm life for children. No specific theoretical framework was adopted *a priori* in developing or conducting the interviews.

Twelve of 19 volunteers were reached and interviewed via telephone in their own home at a time deemed convenient to them. Informed consent was obtained verbally, a method approved by the University of Saskatchewan Research Ethics Board. Interviews were 10–37 minutes long (mean 25 minutes) and conducted between March and April 2016 by a member of the research team (VE), digitally audio-recorded, and transcribed verbatim. Of these, one interview was excluded from analysis due to the family not living on a farm.

Analysis

Thematic analysis of interview data was informed by Interpretive Description method with particular attention to the experiential knowledge and perspective of participants [12]. Analysis was performed by three members of the research team (VE, CT, AC) using NVivo qualitative data analysis software (QSR International Pty Ltd. version 11). The process was led by a member of the research team with methodological training and expertise in qualitative research (AC). Two of the team members performing analysis come from agricultural backgrounds (VE, AC). Transcripts were first open-coded then grouped into relational categories by two team members (VE, CT) and the process was repeated for verification on a sub-set of transcripts by a third team member (AC). Memos and notes were used throughout the process of analysis to deepen the conceptual interpretation. Next the data were examined to develop major themes and team members met to refine the emergent conceptual findings (VE, CT, AC). The final model and thematic categories were presented to the full research team for review and scrutiny. Direct quotations from participants are used to illustrate each aspect of the findings and demonstrate that the analytic interpretations are rooted in the data.

Findings

Summary of findings

Telephone interviews were successfully conducted with 11 farm parents in the Canadian Province of Saskatchewan. Interview participant demographics are presented in Table 1.

Our analysis yielded two key findings:

Table 1. Participant demographics for semi-structured telephone interview of farm parents.

Parent ID	Age	Sex	Born/ raised on Farm	Type of farming operation	Years farming or living on a farm	Number of children	Ages of children	Sex of children
**1**	31–40	Male	Father	Mixed	11–15	3	At least one child age 7–12	Girls
**2**	41–50	Female	Father	Grain	21+	3	At least one child age 7–12	Both
**3**	31–40	Female	Mother	Mixed	16–20	4+	At least one child 6 or under	Both
**4**	31–40	Male	Father	Mixed	21+	3	At least one child 6 or under	Both
**5**	31–40	Female	Both	Mixed	11–15	4+	At least one child 6 or under	Both
**6**	41–50	Female	Mother	Cattle	11–15	1	All children teens	Boys
**7**	31–40	Female	Both	Grain	11–15	2	At least one child 6 or under	Both
**8**	41–50	Female	Father	Grain	21+	2	All children teens	Both
**9**	31–40	Female	Both	Cattle	11–15	2	At least one child 6 or under	Girls
**10**	31–40	Female	Father	Acreage/Hobby	< 5	3	At least one child 6 or under	Girls
**11**	31–40	Male	Father	Mixed	21+	2	At least one child age 7–12	Girls

https://doi.org/10.1371/journal.pone.0198796.t001

First, farm parents engage in a process of assessment of the perceived risks and rewards of bringing children into the farm worksite, with the inherent balancing of tradeoffs or risk-benefit analysis (Fig 1).

Second, multiple factors influence the perceptual 'lens' through which they view this risk-reward tradeoff (Fig 2).

Risks, rewards, and tradeoffs: Perceptions of risk

Parental decision-making involves weighing the pros and cons as if on a balance scale (Fig 1). On one side of this scale sits the hazards perceived by the parent(s): inadequate supervision,

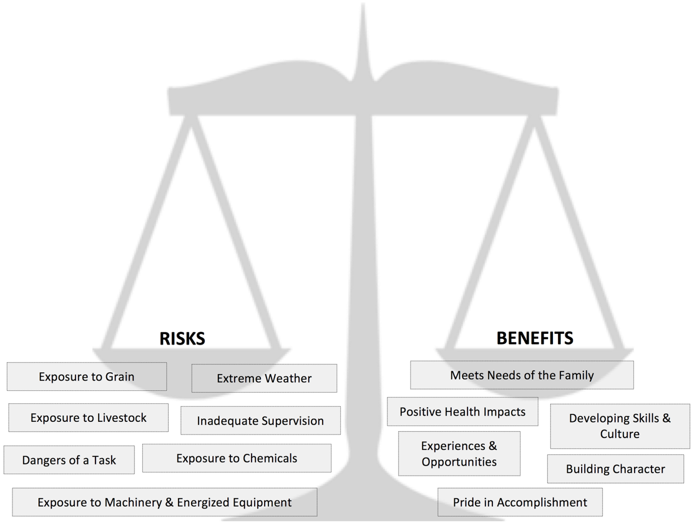

Fig 1. Balance scale model of the risks and benefits of bringing children into the farm work environment.

https://doi.org/10.1371/journal.pone.0198796.g001

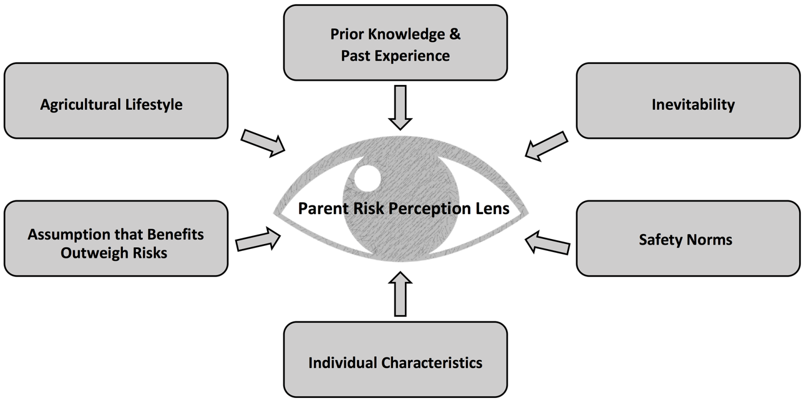

Fig 2. Risk-perception 'lens' model of factors influencing farm parents' decision to bring children into the farm work environment.

https://doi.org/10.1371/journal.pone.0198796.g002

task-related dangers, extreme weather, and exposure to farm hazards such as grain, equipment and machinery, livestock, and chemicals. Counterbalancing this are the parent-perceived benefits: meeting family needs for childcare, labour, and family time; unique opportunities and experiences; building character; learning skills and acquiring cultural values; pride in accomplishment; and positive health impacts.

Inadequate supervision was seen as a risk and a reason not to bring children into the farm environment:

> "...when we're really busy...when we're tired [or] don't have time to properly watch them, they're not there" (Parent #1)

Parents also described a potential mismatch between the **dangers of a task** and the developmental ability of children to safely navigate any environment where those dangers are present:

> "anything that would be ...beyond their capabilities, we're not going to have them around" and "it's specific to what... your child can handle." (Parent #2)

This idea of a potential mismatch coincides with parent's relating that as children grow and develop, so will their abilities to mitigate hazards in their environment. For example:

> "If there's...any equipment inside of a bin then they've never been in there...I don't know at what age they'll be old enough to do that but it's certainly not now. And working up on ladders, heights, confined spaces and places like that they...have no place in there at this point either" (Parent #12)

Residing in a region where winter temperatures can regularly be below -40 degrees C, **extreme weather** was cited as a risk to children:

> "...we don't bring the kids out if it's cold...". (Parent #10)

Exposure to several farm-specific hazards was also named by parents as being a consideration that would limit their bringing children into the farm worksite. *Grain* engulfment was named as a concern, and particularly in light of recent news reports of child fatalities by grain engulfment:

> "any child could be at risk for. . . being smothered by grain if they're playing in an unsafe area" (Parent #7)

Several parents alluded to these events in their comments not only about grain as a risk, but also in terms of using the events as opportunities to discuss farm safety as a family.

While riding in machinery with parents was described frequently as a situation where children might accompany parents into the farm work environment, contact with *machinery and energized equipment* were also named as a hazard:

> "[we]. . .really prefer our younger children [to] not be around grain augers. . .because things can change so quickly and you have to zip over to the [tractor] cab and turn the PTO [power-take-off device] off. . ." [authors' note: running machinery like augers and PTOs present a risk for entanglement of clothing and amputation of limbs] *(Parent #5)*

Similarly, working with machinery to distribute fertilizer or pesticide *chemicals* was identified as a reason not to bring children along:

> ". . .we don't take them when. . . spraying. . . or anhydrous, they're not allowed around. . .any of the chemicals." (Parent #7)

Livestock were also identified as being unpredictable and occasionally aggressive in a way that requires adult supervision:

> "The cow chores, just depends on what's going on. . .sometimes we're out there for quite a bit of the day. . .that's not really a safe place for the kids. . .if your attention is diverted for any length of time at all." (Parent #10)

Although collectively the list of hazards seems comprehensive, not all parents identified each of the risks, and many prioritized the various risks differently. The variability in the scale or intensity of risks that were described by the parents speaks to the variability of risks and perceptions.

Risks, rewards, and tradeoffs: Perceiving the benefits

While parental consideration of farm hazards was in general insightful and detailed, the identified benefits of bringing children into the farm work environment were many and passionately described, centering on a child's development, values, and family life. In terms of the **needs of the family** unit, bringing children along while parents work can help achieve goals for supervision and farm labour:

> ". . .leaving them on their own is not an option. And as they got older. . .they were helping" (Parent #3)

Tasks assigned to children included gathering eggs, feeding animals, and moving vehicles:

> "He is a helper and he can drive the tractor. . ." (Parent #6)
>
> ". . .we'll have to drench a calf or something or get one in the barn and I just can't do it by myself some days." [authors' note: drenching a calf is administering a feeding tube, which calves often resist and struggle] *(Parent #6)*

Some tasks were far simpler, but not trivial in the farming context and considered vital from the parents' perspectives:

"...come on I need your help, you gotta open gates for me..." (Parent #6)

Parents described that bringing children along also provides an opportunity for the family to spend quality time together in spite of busy work schedules. For example:

"...to be together. I think it's more about the family unit... because we have a job to do and they're coming along... because we're together doing something as a family ..." (Parent #6)

"I have very little time with my kids so if they're able to come out and participate in work together, it's a good opportunity for us to reconnect." (Parent #12)

Farm parents also described how farm life and exposure to the farm work environment affords children enriching **opportunities and experiences**:

"they...have a lot of opportunities and experiences that children who are being raised in a city wouldn't have" (Parent #2)

Farm parents likewise described that these experiences may provide advantages not generally available to non-farm kids in terms of preparation for the demands of adult life:

"...when it comes time for them to have a driver's license and handle a vehicle they're really gonna have a leg up because the reality is that we live on a grid road and that's always trickier driving on a grid road. And in winter time and things like that; they'll be able to handle a vehicle really well because they've had a lot of practice already at it just out in the field." [authors' note: a grid road is an unpaved municipal rural road] *(Parent #12)*

Overlapping with the idea of children's development and capacity, parents identified **opportunities** for building children's skills and awareness around livestock:

"...we preach to our girls all the time 'don't walk around the cows and calves', especially as children, just stay out of there." (Parent #10)

"...as you walk through or you take the kids checking cattle with you, you'll say to them 'you see what that cow is doing with her ears?' or 'do you see what that cow is doing with her foot...she's trying to tell you something'..." (Parent #5)

Parents described that these advantages extend to developing desirable traits and '**building character**', including developing work ethic and responsibility and reducing entitlement:

"...our kids start their day before 7. They get up and get on the bus by 7:45. It's about a 45–50 minute ride & same thing coming home. It's a different lifestyle, but all that builds into the character of being on the farm..." (Parent #2)

This benefit was described essentially as a transfer of values between parents and children, who learn by example by absorbing these values within the farm environment:

"...they're there participating, learning, and learning work ethic, observing and you teach them as you go" (Parent #5)

"…to know the value of a dollar, to be accountable, they have their own jobs that they have to do and they see what happens if they don't do them…" (Parent #1)

'Building character' is closely related to the next benefit identified by parents: '**developing skills and culture**', wherein children are learning specific skills and developing an appreciation for farm culture (Fig 1). If children have a natural affinity for learning about 'the world around them' then having that 'world' be a farm has an impact on their learning:

"…they're interested. They wanna see what's going on, they wanna be a part of it…that's farm life" (Parent #1)

"there's a lot of value in knowing how things work, why things happen…if the kids are able to see from start to finish they learn a lot" (Parent #1)

Parents described an affection for farm life, involvement with seasons and lifecycles, and in some cases a sense of agriculture fitting into a larger whole; this led to a natural inclination to share this with their children:

"it's just a natural thing to bring your children into that culture…that way of life…gathering eggs…raising chickens…pigs, having cattle, seeing their new babies…[it's all] part of it & you don't want them to miss it." (Parent #6)

In addition to the benefit of learning specific skills such as winter driving, safe performance of farm tasks was acknowledged as needing 'hands-on' demonstration and training:

"That's actually why I think it's important to have the kids with us doing something, is for the safety part. For us to show them by example…how to do things and to point out the consequence…" (Parent #10)

Involvement in farm work, including experiencing the sacrifices and hardships involved in hard work (however slight), was seen to develop not only character but also **pride in accomplishmen**t (Fig 1):

"…they can help too, like they're a big help and they find accomplishment within doing that…" (Parent #3)

Parents described that this sense of accomplishment can in turn foster an appreciation for family values around contributions to the whole and the rewards of collective effort:

"I think it creates a sense of family and striving toward a common goal, you know because they're out there and they're helping daddy or helping mommy. And kids flourish in that environment." (Parent #5)

Lastly, parents mentioned '**positive health impacts**' for their children as a benefit of farm work, embodied in the health-enhancing effects of time spent outdoors, physical work, and interaction with family members and goal-oriented activities:

"…physical activity…they're out carrying food and water and they're participating, walking around the yard and doing things like that… an active lifestyle is important to lead our

children so they have those health benefits carrying on throughout their lives. . ..” (Parent #12)

The benefit of physical, outdoor activities was given particular importance in an age where sedentary activities and ‘screen time’ have become more common:

“. . .if you kept them in the house all the time. . . there’s risk in that! If they’re always on the computer there’s always risk within that.” (Parent #3)

Risks, rewards, and tradeoffs: A balancing act

Variation in what parents identified as an unacceptable risk or hazard suggests that there are factors that can ‘tip the scales’ and favour one decision over another. Given that parents are constantly weighing their options in any given set of circumstances, what influences their decision making?

Influencing factors & the risk assessment lens. How a parent’s decision-making process ‘tips the scales’ to either bring or not bring a child into the farm worksite depended on their perception of the risk-benefit tradeoff. Their perception in turn depended on their ‘perceptual lens’, conceived here as an eye that forms the pivot point of their decision-making balance scale (Fig 1). A parent’s perceptual lens was influenced by many factors: the agricultural lifestyle as a way of life, parents’ prior knowledge and past experience, assumption that benefits outweigh the risks, individual characteristics of the children, safety norms, and a sense of inevitability or conversely control over the outcome (Fig 2). These influencing factors did not all have the same weight, as their weights can vary by occasion, by context, by scenario, and between parents or families.

A primary influencing factor in the parents’ risk perception lens was the exceptionalism of the **agricultural lifestyle** (Fig 2). Beyond just the job title ‘farmer’, parents described farming as a way of life, a way of interacting with the natural world and with each other as family members. Farming was often presented in contrast to other occupations where the time commitment is lower and the work is less embedded in all or most aspects of an entire family’s life:

“. . .farming is a lifestyle. It starts from the time that you wake up, pretty much to the time that you go to bed. . .kids on the farm are a part of it because that’s just what we do. We eat, breathe, sleep the farm. . .that’s the nature of what we do. . .it does allow for grabbing those moments of time together. . . sitting in a combine and you’re on a long swath and having a great conversation with one of the kids. . .It’s just a part of what we do and. . .I don’t see how farming can work if you can’t include your family as part of it. Because it’s just not a 9 to 5 kind of an occupation. . .” (Parent #2)

This notion of a unique agricultural lifestyle includes a sense of identity tied into one’s heritage and culture, especially on family farms passed down through generations. The ability to spend time with family members while performing farm work fills a basic parenting need (supervision) and allows for instilling values. Building on these benefits, adherence to the cultural values around the agricultural lifestyle preserves family ideologies and family traditions, with an ultimate goal to perpetuate this lifestyle and reproduce family values in a new generation of farmers within the family:

“Ultimately our goal is that one of our kids is gonna take over the farm, we hope. So you want them involved in the lifestyle.” (Parent #1)

In this context, the choice not to include children in farm activities (including exposure to the farm work environment) would deprive them of this opportunity and threaten the values held by the family.

Parents were also influenced by their **prior knowledge and past experience** on the farm, of their children, and of injury or traumatic incident in their communities (Fig 2). The cumulative effect of parents' personal experience and knowledge about risks can have a strong effect on their decisions, with personal experience tending to drive perception of potential consequences:

> ". . .we've never had anybody get hurt! So. . .but we're pretty careful too. . ." (Parent #11)
>
> ". . .last summer. . . some of the very public accidents that happened. . . we very much used those situations to talk to our kids about the different risks. . . such a tragic situation and we just had a lot of conversations around kind of the dinner table. . ." [authors' note: 'public accidents' refers to local media reports of farm child fatalities in these communities] *(Parent #2)*

Familiarity with farm hazards may result in habituation and reduced risk perception 'getting used to it' even if real risk remains the same. This is particularly evident with parents who grew up on a farm and have already acquired ease with navigating farm hazards:

> "[daughter] has been involved more in driving vehicles. It just sort of started out that [way]. . .which was totally a shock to me that this happened on the farm but. . .my husband grew up on the farm and he was like 'young kids are around machines. . .they drive machines. It's something that normally happens'. And so he's always been the one that's said 'I was way younger when I was driving'. . ." (Parent #8)

When rewards of a decision are mixed with risks as in the balance scale (Fig 1), risks can be seen as lesser than they actually are. The resulting influence on parents' decision making is an **assumption that the benefits outweigh the risks** (Fig 2). Given that there may be a compromise in weighing two options, parents are left to choose the option that they perceive to be the *least* detrimental, and the 'correct' option will be the one that they perceive to have the best overall outcome for the child:

> ". . .living an active lifestyle. . .means you have to go out and do things. . .my belief is. . .the benefits well outweigh the risks and you do everything that you can to reduce and minimize those risks." (Parent #12)

Deciding to bring children along may involve two imperfect options rather than clear benefits; in this case the "least-worst option" might be selected. For example, when child-care options are limited, the perceived choice may actually be between two risks:

> ". . .the odd time it would be because something really has to get done and . . . uh . . . the kids have to come along for their own safety right; we don't want them alone at the house either." (Parent #10)
>
> "my husband was in a bit of a bind and didn't have a driver and it was easier to move 2 vehicles instead of just one so he did agree to let my son [come along]. . ." [authors' note: this refers to a situation where it is easier to have the child help move a vehicle when two vehicles are needed in a work area] *(Parent #10)*

"...they're by themselves a great deal...later I get a text from my son...[who] had made...his supper at 9 [years old] and I'm like, 'you're making scrambled eggs on the stove yourself, hmm!' ... he worked it out so. You know, I guess that's okay." (Parent #8)

Parental decisions are framed in Fig 1 as balancing a tradeoff between risk and benefits, and an assumption of a 'winning' tradeoff can influence these decisions. While decisions were not always easy, many parents described 'common sense' as a guiding principle in determining whether to bring children into the farm work environment: *"I think common sense has to prevail."* However, while 'common sense' was implied to be a universal standard, the definition of 'common sense' was inconsistent between parents, who differed in what was permitted, and at what stage.

For instance, parents' decision-making may also be influenced by **individual characteristics** of both the parent and the child (Fig 2). Children's gender, interest in farm activities, risk-taking/risk-aversion orientation and ability or stage of development can also influence parents' decision making. In terms of ability, one parent described how a child with a disability required different considerations for safely entering the farm work environment, and that there were more restrictions on the situation and hazards present. In terms of development, many parents described how some hazards (machinery, moving grain) would not be appropriate for small children, but that their children would grow into doing that type of work. Risk orientation was described in terms of adherence to safety rules and a relative propensity for individual children to take risks:

"...my son is quite mature...he's an only child...he's really safety smart. For a 13 year old, he's not really a major risk-taker. He's really fairly cautious." (Parent #6)

The parents' individual characteristics such as gender and age (Table 1) may also influence their decisions:

"I have some friends that don't trust their husbands with their children. Meaning like they'll be out without a jacket and it's minus 20 and then no toque. . .." (Parent #6)

Not all parents have the same orientation towards safety or risk assessment, and they may have divergent attitudes towards the importance, effectiveness, and desirability of prevention; this can be seen in parents' willingness to make substantial investment in farm safety, either in terms of financial or time investment:

"We've taken precautions...like we don't have any overhead power in our yard...it's all been buried for like 20 years now" (Parent #4)

"...with our morning meetings with staff, the kids are around... So then we try to inform the kids before we go out and then once we are out there, we watch them doing stuff and explain..." (Parent #1)

The social construct of **safety norms** may influence parents in subtle ways. The social milieu formed by the rural agricultural community, immediate and extended family can influence what is considered 'normal' and 'safe'. As described in 'prior knowledge and experience', a parent who grew up on a farm may have a different perception of risks and the effectiveness of mitigation strategies. Safety norms are not static, and can show substantial evolution over time, as exemplified by a parent describing generational changes in norms for children as tractor passengers:

"...I know they always wanna come and ride on the fender or something like that. And...when I was 5...ya, that seemed to be the norm. But nowadays it's not practical." (Parent #4)

Perceptions of what is safe can also differ between rural and urban environment, and parents were perceptive in identifying that their location informs their view of 'safety':

"...you see situations [in the city] where kids are walking down the street and they're small...and it's like 9:30 at night. And there's no adult with them and you think how on earth would they let their kids do that?" (Parent #2)

A belief in the **inevitability** of injury and traumatic incidents was expressed in many of the interviews, and thus positioned as being influential on decision-making (Fig 2). The somewhat fatalistic notion that "accidents happen no matter what" may free parents from a paralysis of overprotection and provide some perspective on the unrealistic expectation of preventing all possible harm:

"....there's just no way to avoid all risk." (Parent #12)

Related to this idea of inevitability is the idea that some exposure, education, and knowledge about risks can help protect against future harm, since children will be equipped with safe practices and thus better prepared to deal with the hazards:

"...they learn to be in situations where they have to watch and they learn certain skills that will help them later on to identify problems and threats..." (Parent #1)

Integrated model of parents' decision making. The present study delivered two main concepts in its findings: the 'risk-benefit balance scale' presented in Fig 1 and the 'parents risk perception lens' presented in Fig 2. The combination of these findings results in a comprehensive model demonstrating the complexity of parents' decision making and illustrates how it can vary over time, by situation, and between parents or households (Fig 3).

As shown in the integrated model, parents' risk-benefit calculations are ***not*** merely a quantified tradeoff of risks and benefits, but is also influenced by various degrees and by several factors: the agricultural lifestyle as a way of life, parents' prior knowledge and past experience, assumptions that benefits outweigh risks, individual characteristics of the parents and children, safety norms in the social milieu, and a feeling of inevitability.

Discussion

This study aimed to better understand farm parents' views on the benefits and challenges of raising children safely on a family farm, as well as their decision-making processes regarding bringing children into the farm worksite. To do this, we conducted 11 semi-structured telephone interviews with parents to describe the influences and considerations that play into these decisions. This study provides a description of how some farm parents in the Canadian province of Saskatchewan decide when, how, and if to bring their children into farm work environments. Interview themes were used to develop three models illustrating the interacting, continually evolving complexities involved in these parental decisions. The first model (Fig 1) illustrates that parental decision-making involves weighing perceived risks and benefits as if on a balance scale; their perception of the risk-benefit tradeoff is what 'tips the scales' for a decision. The second model (Fig 2) illustrates parents' perceptions that are influenced by their

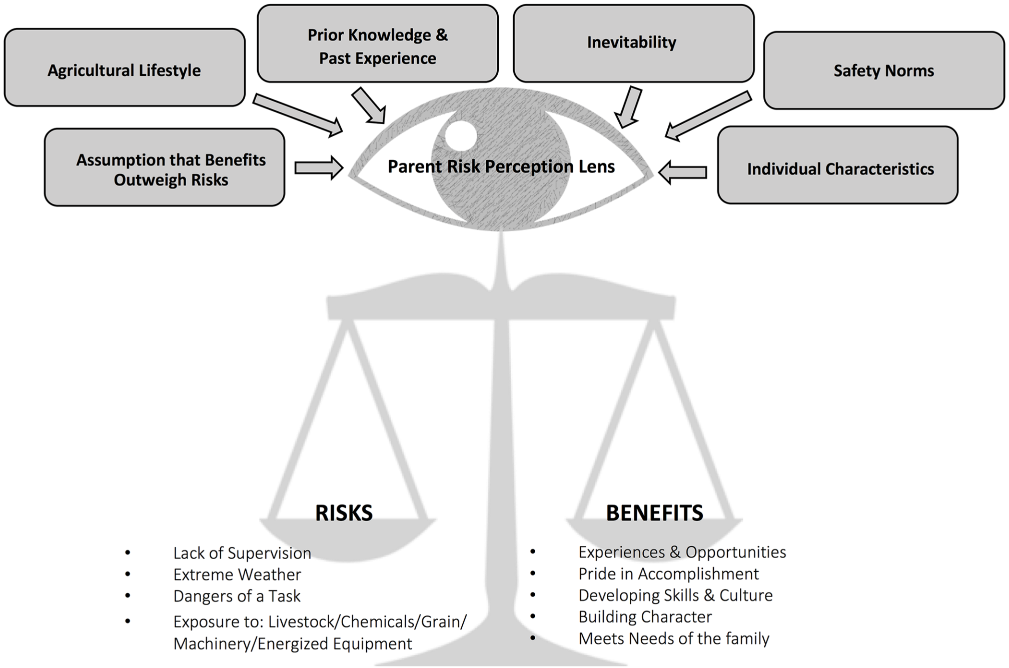

Fig 3. Integrated model of farm parents' decision-making process for bringing children into the farm work environment.

https://doi.org/10.1371/journal.pone.0198796.g003

'perceptual lens', shaped over time by many interrelated factors. The third model provides an integrated illustration of the first and second models, demonstrating the interconnected processes involved in the parents' decision-making. These models provide a deeper understanding into the decision-making processes of these Saskatchewan farm parents and are proposed as a starting point for engaging with farm parents about these issues. Perhaps by listening to their viewpoint we can better inform effective interventions to prevent agricultural injuries to children on farms.

Parental responses indicated their knowledge and awareness of hazards for children on farms, as well as the many benefits of being raised in this environment. The fact that parents are knowledgeable about the hazards on the farm may be indicative of the educational focus of intervention strategies that are often utilized in injury prevention initiatives [13]. The main risks identified by the parents in this study are common injury risk factors for children on farms including: inadequate supervision [4, 14], task-related dangers, extreme weather [15], and exposure to farm hazards such as grain [4], equipment and machinery [4, 16, 17], livestock [4, 16, 17], and chemicals [16]. Although water sources were not identified as a risk factor, drownings are a known cause of pediatric farm injury in Canada [4]. Benefits of farm life have been described less often in the literature, but still acknowledged [8]. These include meeting family needs, developing skills and culture, building character, experiences and opportunities, pride in accomplishment, and positive health impacts [6, 7].

Although parents know about the safety hazards and risks present on the farm, decisions to bring children into the farm work environment continue because to the parents, the benefits outweigh the risks (see Fig 3). This is consistent with **rational choice theory** [18], which suggests that people make decisions that give the greatest perceived benefit based on the information and choices they have. For example, in one scenario, a parent may decide that bringing the child along when doing farm work is safer than leaving the child at home alone and allows

the parent and child to spend time together. Different scenarios may swing the balance of the risk-reward tradeoff in different directions; decisions may be made in situations with varying degrees of stress (for example, during busy harvest, seeding, or calving seasons). Often parents feel they must make an 'imperfect choice', choosing what they perceive to be the least potentially harmful.

The perceived balance of risk and reward depends on the perceptual lens of the parent. It appears that parents/caregivers make decisions about the risks and benefits of bringing children into the farm work site through a complex series of inputs that form a 'parental risk perception lens' (see Fig 2). Factors that influence the perceptual lens include: lifestyle/culture/way of life; prior knowledge and past experience; assumption of benefits outweighing risks; individual characteristics; safety norms; and inevitability. Each of these 'perceptual influencers' differ in weight which can also vary by occasion, scenario, and between parents. **Lifestyle/culture/way of life** are major contributors to the perceptual lens of parents. In agriculture, farm families work together and play together and parents often alluded to the fact that farming is not just a job, it is a way of life and part of their personal identity. This is consistent with cultural theory [19] and the agrarian myth [20] wherein agriculture serves a larger purpose of preserving ideologies and traditions vital to maintaining the farming industry (and family) for future generations. Specifically, a parental choice to not include children in farm work activities threatens the values fundamental in farm family culture.

Prior knowledge and **past experience** also play a key role in the development of one's perceptual lens, and can have a cumulative effect on parental decisions. For example, a parent who was raised on a farm and is familiar with farm hazards and safety issues may have very different views of parenting in the farm environment than a parent who was raised in an urban environment. In addition, parents may see the positive benefits of farm life as certain and the risks uncertain, consistent with pseudocertainty effect [21]. When rewards are mixed with risks, risks can be perceived as lesser than they actually are, which leads to the next important influencer on one's perceptual lens, the **assumption of benefits outweighing risks.**

As with rational choice theory, parents may feel they need to weigh out two or more risky scenarios to decide which is the least potentially detrimental choice. Parents often indicated their reliance on 'common sense' to make such decisions; however, common sense is not a universal construct. **Individual characteristics** of the parents and of the child affect the perceptual lens of the parents and their decision-making that is related to the parents' and the child's gender, risk-taking level, desire/interest and the developmental ability of the child. Another key influence on this perceptual lens is **safety norms** that can vary between parents, generations, families, geographic background, and children. Lastly, an important contributor to the parents' perceptual lens is the somewhat fatalistic view of **inevitability** where parents expressed their perception that 'accidents happen' no matter what and they prefer to let their children live by experiencing and not 'keeping them in a bubble'.

The present findings are consistent with those of a previous study involving interviewing adult male-female farm couples [5]. Those authors described farmers as having strong awareness of hazards, but gaps between knowledge and behaviour arise from influencing factors. For example, the latter include work constraints, personal experience, and perceived vulnerability as well as gender, with women tending to identify more opportunities for safety behaviours. The notion of a 'safety calculation' between benefits and risks has previously been reported [5].

Implications

An overarching motivation for this study was to provide a knowledge base that could contribute to the future development of appropriate interventions to prevent farm injuries to children

that would be more acceptable to farm parents, families, and communities. Previous strategies for injury prevention have considered the importance of three main aspects: Engineering (environmental changes to remove or reduce a hazard), Enforcement (policy/regulations), and Education (awareness to promote behaviour change) [22]. With few exceptions, educational strategies can be successful in increasing knowledge of hazards, shown by parental awareness. However, injury prevention initiatives that rely solely on any one strategy alone are less likely to be effective in bringing about substantial positive changes in safety outcomes for both children's safety [1], and general farm safety [23].

The development of interventions needs to focus on behavioural changes that remove or reduce the exposure of children to hazards in the farm work area. Effectively motivating behavioural change requires credibility on the part of the messenger, and for the message to demonstrate understanding of the culture of the receiver. In this case, it will be important to acknowledge the fundamental beliefs and values of a family and benefits of farm life and of age-appropriate engagement in farm work for the parents to really embrace the intervention [1]. In the present study, several parents asked the interviewer whether she came from an agricultural background and appeared to be reassured when they were informed that they were speaking to someone with that background. This may reflect either a decreased fear of judgement or a comfort in shared understanding/background, but in any case should be a consideration when approaching farm community collaborators and developing farm child safety interventions. Appropriate next steps include further engaging in dialogue with farm parents, either to co-create the intervention or to get in-depth focus group input on prevention strategies using a community-based or participatory approach.

With the decision-making framework as a starting point, there can be acknowledgment of both constraints and benefits, and advice can be centred around a harm reduction approach which reduces exposure to the highest risk conditions and mitigates risk of exposure in 'low-to-medium-risk' conditions. An example starting point may be re-framing the North American Guidelines for Children's Agricultural Tasks (NAGCAT) guidelines [3] to demonstrate how families reap the benefits of farm life throughout the developmental stages while reducing risk of child injury.

Strengths and limitations

By interpreting the interviews with farm parents, this study provides novel insight into how these Canadian farm parents make decisions about bringing their children into farm work environments. Our model provides the first composite of decision-making concepts and theory in a farming context, providing a unique framework for application in future intervention development. In terms of methodological strengths, this study kept a detailed audit trail and triangulated data from two sources (written surveys and telephone interviews). Since the written responses and interview responses gave coherent and consistent information with no inconsistencies in themes/subthemes between data sources, the findings can be considered consistent, credible, and robust. Team members met regularly to review codes, concepts, and themes, thus ensuring confirmability of results. We consider our interpretation to be trustworthy, given the team members' expertise in qualitative methods (AC), personal experience as a member of a farm family (VE, AC), and history of research in child farm injury (WP, BM). This sample may not be representative of all farm parents; it is not the intention of qualitative research to represent the whole population, but rather to provide in-depth insight on an issue. Although the sample size of 11 was not large, we reached saturation of themes and maintained coherence with the developed model with this sample. Study limitations also warrant comment. Since other geographic regions cultivate other products and therefore have other risks,

the focus on the rural Saskatchewan context may limit transferability to other regions and farm types. Given that the safety of children has the potential to invoke defensiveness, the sample may have under-represented parents who felt more defensive, who had experienced a serious injury on their farm, or who had less of a safety orientation. Likewise, social desirability bias may have impacted the candor of responses in terms of reluctance to disclose unsafe behaviours and the decision making that led to them. All questions were asked within the context of a child injury study and written responses embedded within a child injury questionnaire; this may have influenced the nature of the responses as respondents were 'primed' to this topic.

Conclusion

This qualitative study provides in-depth information to better understand the conditions under which children are exposed to farm hazards. It may also provide requisite information from which to engage with parents in developing effective interventions. Moving forward, the complex nature of this issue will call for a thoughtful and integrated approach that considers parental views as integral to the solutions. The findings may serve to bridge a gap between health promotion efforts and these parental views, an ultimately help to foster practical solutions that are acceptable to the farm community.

Acknowledgments

The authors wish to thank the administrators from participating school boards and schools, as well as the parents and families who contributed. We thank Wes Dunham for his contributions in coding of the initial written survey questions. The present study was conducted as part of the larger Saskatchewan Farm Injury Cohort Study; this study team also includes: William Pickett, Queen's University (Principal Investigator, pickettw@queensu.ca), James A. Dosman, University of Saskatchewan (Co-Principal Investigator), Josh Lawson University of Saskatchewan, Robert Brison, Queen's University, Louise Hagel, University of Saskatchewan, Don Voaklander, University of Alberta, Catherine Trask, University of Saskatchewan, Andrew Day, Kingston General Hospital, Lesley Day, Monash University, Barbara L. Marlenga, Marshfield Clinic Research Foundation, Niels Koehncke, University of Saskatchewan, and Valerie Elliot, University of Saskatchewan.

Author Contributions

Conceptualization: Valerie Elliot, Allison Cammer, William Pickett, Barbara Marlenga, Joshua Lawson, James Dosman, Louise Hagel.

Data curation: Valerie Elliot, Allison Cammer.

Formal analysis: Valerie Elliot, Allison Cammer, Catherine Trask.

Funding acquisition: William Pickett, Barbara Marlenga, Joshua Lawson, James Dosman, Niels Koehncke.

Investigation: Valerie Elliot.

Methodology: Valerie Elliot, Allison Cammer, William Pickett, Catherine Trask.

Supervision: Allison Cammer, William Pickett, Catherine Trask.

Validation: Allison Cammer, Catherine Trask.

Visualization: Valerie Elliot, Catherine Trask.

Writing – original draft: Valerie Elliot, Barbara Marlenga, Catherine Trask.

Writing – review & editing: Valerie Elliot, Allison Cammer, William Pickett, Barbara Marlenga, Joshua Lawson, James Dosman, Louise Hagel, Niels Koehncke, Catherine Trask.

References

1. Wright S, Marlenga B, Lee BC. Childhood agricultural injuries: An update for clinicians. Current problems in pediatric and adolescent health care. 2013; 43(2):20–44. https://doi.org/10.1016/j.cppeds.2012.08.002 PMID: 23395394
2. Brison RJ, Pickett W, Berg RL, Linneman J, Zentner J, Marlenga B. Fatal agricultural injuries in preschool children: risks, injury patterns and strategies for prevention. Canadian Medical Association Journal. 2006; 174(12):1723–6. https://doi.org/10.1503/cmaj.050857 PMID: 16754900
3. The North American Guidelines for Children's Agricultural Tasks (NAGCAT): Marshfield Clinic Research Foundation; [2017]. Available from: https://www.nagcat.org/nagcat/.
4. CAIR. Agricultural fatalities in Canada, 1990–2008. Edmonton, Alberta: 2011.
5. Green K. Farm health and safety: Rural couples' beliefs and practices. Journal of agricultural safety and health. 1999; 5(1):83.
6. Ashida S, Heaney CA, Kmet JM, Wilkins JR III. Using protection motivation theory and formative research to guide an injury prevention intervention: increasing adherence to the North American Guidelines for Children's Agricultural Tasks. Health promotion practice. 2011 May; 12(3):396–405. https://doi.org/10.1177/1524839910362034 PMID: 21518923
7. Zepeda L, Kim J. Farm parents' views on their children's labor on family farms: A focus group study of Wisconsin dairy farmers. Agriculture and Human Values. 2006 Mar 11; 23(1):109–21.
8. Lee BC, Bendixsen C, Liebman AK, Gallagher SS. Using the socio-ecological model to frame agricultural safety and health interventions. Journal of agromedicine. 2017 Oct 2; 22(4):298–303. https://doi.org/10.1080/1059924X.2017.1356780 PMID: 28762886
9. Nilsson K. Parents' attitudes to risk and injury to children and young people on farms. PLoS one. 2016; 11(6):e0158368. https://doi.org/10.1371/journal.pone.0158368 PMID: 27362751
10. Pickett W, Day L, Hagel L, Brison RJ, Marlenga B, Pahwa P, et al. The Saskatchewan farm injury cohort: rationale and methodology. Public health reports. 2008; 123(5):567–75. https://doi.org/10.1177/003335490812300506 PMID: 18828411
11. Pickett W, King N, Trask C, Michaelson V, Marlenga BL, Hagel L, et al. Factors related to self-perceived health in rural men and women. Journal of agromedicine. 2015; 20(2):178–87. https://doi.org/10.1080/1059924X.2015.1010063 PMID: 25906276
12. Thorne S. Interpretive Description. Walnut Creek, CA: Left Coast Press; 2008.
13. Hartling L, Brison RJ, Crumley ET, Klassen TP, Pickett W. A systematic review of interventions to prevent childhood farm injuries. Pediatrics. 2004; 114(4):e483–e96. https://doi.org/10.1542/peds.2003-1038-L PMID: 15466075
14. Morrongiello BA, Pickett W, Berg RL, Linneman JG, Brison RJ, Marlenga B. Adult supervision and pediatric injuries in the agricultural worksite. Accident Analysis & Prevention. 2008; 40(3):1149–56.
15. Myers J. Special Analysis of Data From the National Institute for Occupational Safety and Health (NIOSH) Traumatic Injury Surveillance of Farmers Survey. National Institute for Occupational Safety and Health, Morgantown, WV. 1995.
16. Hard DL, Myers JR. Fatal work-related injuries in the agriculture production sector among youth in the United States, 1992–2002. Journal of agromedicine. 2006; 11(2):57–65. https://doi.org/10.1300/J096v11n02_09 PMID: 17135143
17. Hendricks K, Goldcamp E. Injury surveillance for youth on farms in the US, 2006. Journal of agricultural safety and health. 2010; 16(4):279–91. PMID: 21180351
18. Blume L, Easley D. Market competition and selection. The New Palgrave Dictionary of Economics. 2008; 5:296–300.
19. Oltedal S, Moen B-E, Klempe H, Rundmo T. Explaining risk perception: An evaluation of cultural theory. Trondheim: Norwegian University of Science and Technology. 2004; 85(1–33):86.
20. Kelsey TW. The agrarian myth and policy responses to farm safety. American journal of public health. 1994; 84(7):1171–7. PMID: 8017549
21. Tversky A, Kahneman D. The framing of decisions and the psychology of choice. Environmental Impact Assessment, Technology Assessment, and Risk Analysis: Springer; 1985. p. 107–29.

22. Christoffel T, Gallagher S. Injury prevention and public health: practical knowledge, skills, and strategies: Jones & Bartlett Learning; 2006.

23. Hagel LM, Pickett W, Pahwa P, Day L, Brison RJ, Marlenga B, et al. Prevention of agricultural injuries: an evaluation of an education-based intervention. Injury prevention. 2008; 14(5):290–5. https://doi.org/10.1136/ip.2008.018515 PMID: 18836044
